# Unusual presentation of oral pyogenic granulomas: a review of two cases

**DOI:** 10.1002/ccr3.1435

**Published:** 2018-02-27

**Authors:** Ramesh Parajuli, Sushna Maharjan

**Affiliations:** ^1^ Department of Otorhinolaryngology Chitwan Medical College Teaching Hospital P.O. Box 42, Bharatpur 10 Chitwan Nepal; ^2^ Department of Pathology Chitwan Medical College Teaching Hospital P.O. Box 42, Bharatpur 10 Chitwan Nepal

**Keywords:** Extragingival site, pyogenic granuloma, tongue, upper lip

## Abstract

Pyogenic granuloma is a benign vascular neoplasm of the oral cavity that usually presents as a small nodular lesion, the gingiva being the commonest site. Occasionally it occurs at uncommon sites with unusual sizes. Here we describe two cases of oral pyogenic granulomas that had an unusual presentation.

## Introduction

Pyogenic granuloma (PG), also known as lobular capillary hemangioma, is a benign vascular neoplasm 1. It results from inflammatory hyperplasia of mucosa or the skin. Its name is a misnomer; neither it is related to pus formation nor composed of true granuloma histologically 2.The neoplastic growth of PG is supposed to be a response to various stimuli such as chronic localized irritation, trauma, hormones, and drugs. Its occurrence in the oral cavity is not uncommon, and poor oral hygiene is considered to be the precipitating factor. It is more common in females**,** implicating the possible effects of female sex hormones on blood vessels. Its peak incidence occurs in the second and fifth decade of life. Gingiva is the most commonly affected site for oral PG followed by tongue, hard palate, lip, buccal mucosa, and the floor of the mouth. Characteristically it presents as a small pinkish soft tissue swelling, the size ranging from few millimeters to a few centimeters. The mass may have a pedunculated or sessile base and is usually nontender, but it can bleed on touch. This report describes two unusually presented PGs of extra gingival sites.

## Case Reports

### Case 1

A 26‐year‐old female presented with the complaint of painless and progressive swelling on the dorsum of the tongue for 1 year. It was associated with slight difficulty in swallowing and speaking. However, it was not associated with bleeding. There was no history of fever, cough, or significant weight loss. She denied any history of trauma. There was no history of similar swellings in other body parts. Her medical and surgical history was not significant.

**Figure 1:**
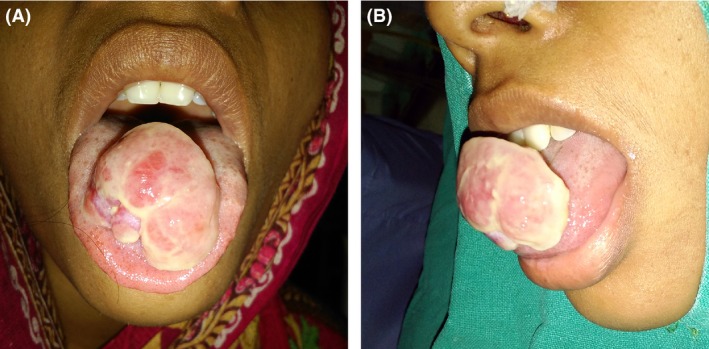
Gross appearance of the lesion arising from dorsum of tongue (A) Front view (B) Profile view.

**Figure 2:**
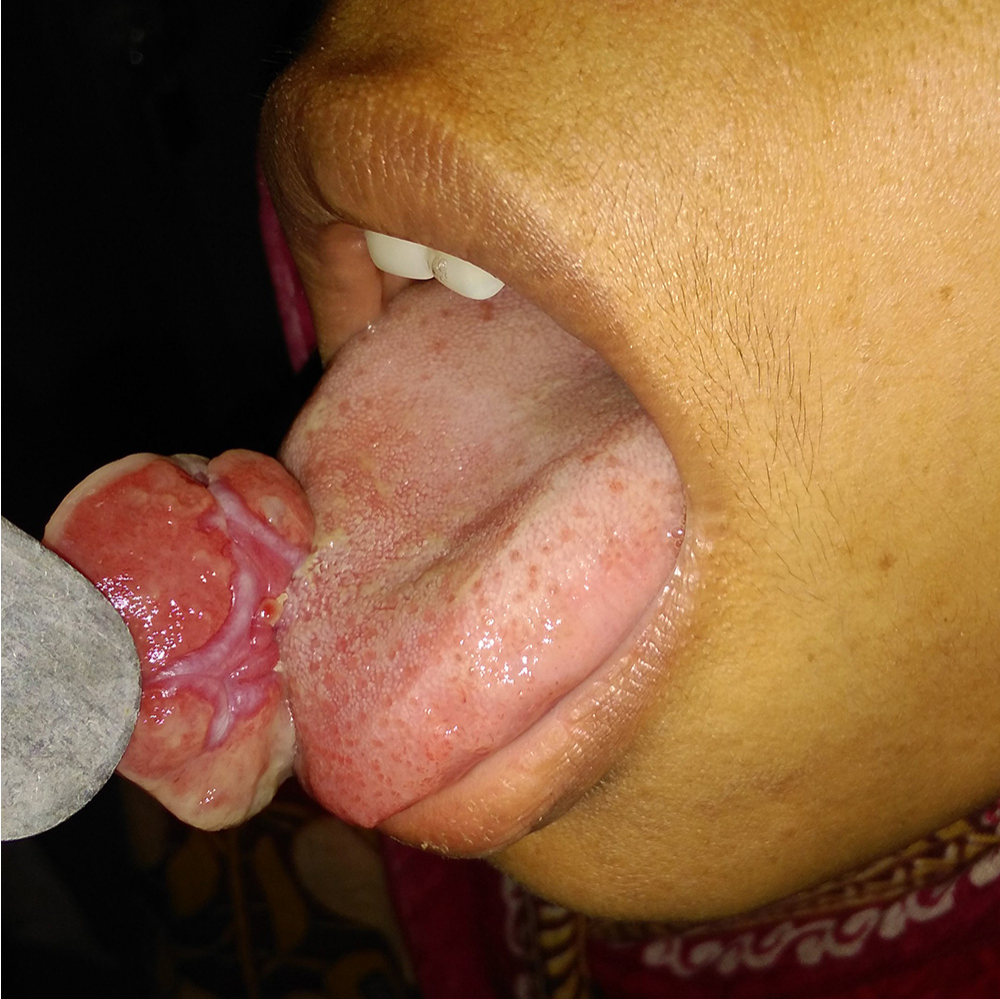
Photograph showing the pedunculated base of the lesion on the dorsum of anterior tongue.

**Figure 3:**
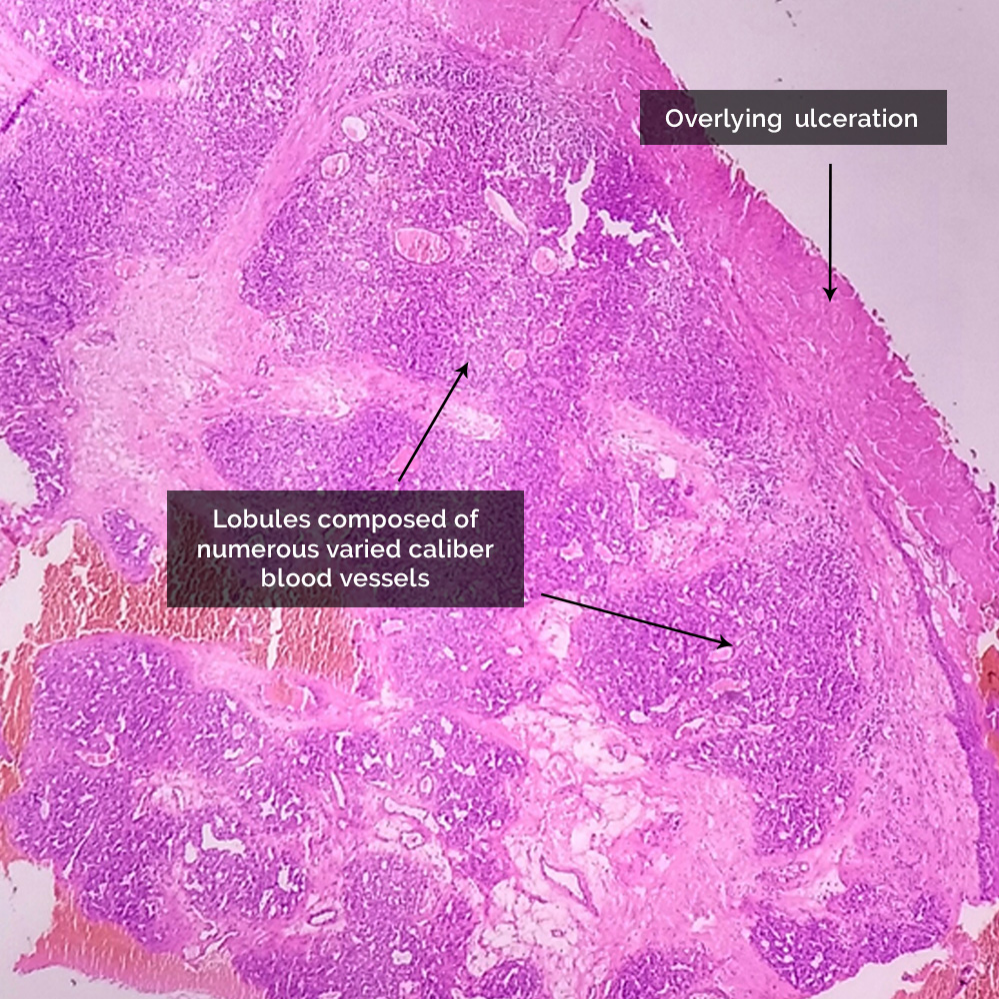
Histopathological examination showing lobules composed of numerous varied caliber blood vessels with an overlying ulceration. (Hematoxylin and eosin stain, ×10).

On examination there was a single, pale to pinkish exophytic growth 2.5 × 2 cm in size, arising from the dorsum of anterior tongue with a pedunculated base (Figs. 1 and 2). It was soft, nontender with a smooth surface that did not bleed on touch. There was no pus discharge from the lesion. Tongue mobility was normal. Her oral hygiene was poor. There was no cervical lymphadenopathy. Based on the history and the physical findings, hemangioma and papilloma were considered as the differential diagnoses. Routine investigation including complete blood count, coagulation profile, serology, and biochemistry were within normal limits. Excisional biopsy was performed under local anesthesia. The histopathological examination(HPE) revealed multiple lobules of capillaries of varying caliber lined by endothelial cells, with an overlying stratified squamous epithelium showing focal ulceration. This was consistent with the diagnosis of pyogenic granuloma (Fig. 3).

### Case 2

A 15‐year‐old girl was brought from a remote village with the complaint of painless and progressive swelling arising from the upper lip for 4‐month duration. It bled occasionally, especially when touched. Her parents were mainly concerned about the cosmetic problem caused by the lesion. Her past medical and surgical history were not contributory. She denied any history of trauma to the upper lip. There was no palpable cervical lymphadenopathy.

**Figure 4:**
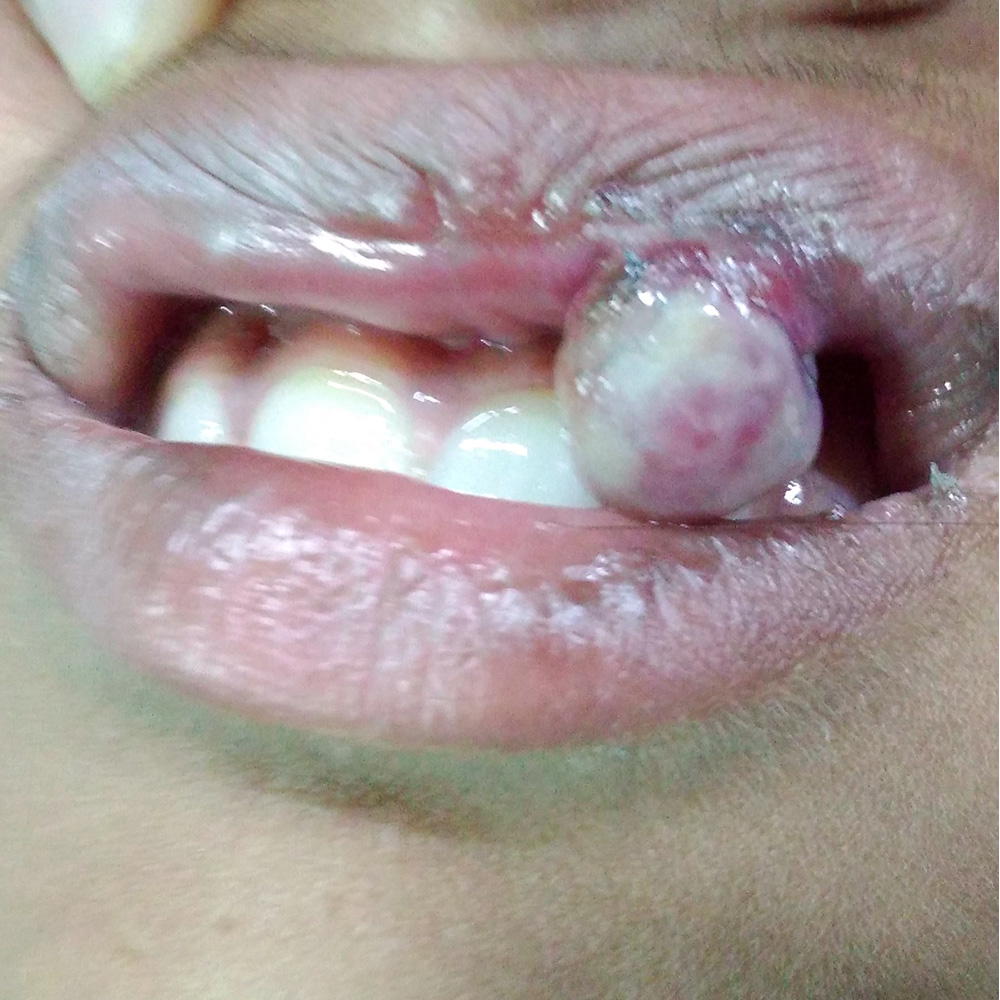
Pinkish, nodular swelling with sessile base arising from the mucosa of upper lip.

On examination, there was a single pinkish mass with a sessile base (Fig. 4),0.5 × 0.5 cm in size, arising from the upper lip. It was mildly tender with minimal bleeding on palpation. She had poor oral hygiene. A provisional diagnosis of hemangioma was made. Routine preoperative investigation, including coagulation profile, was within normal limits. Punch biopsy was taken from the lesion. The HPE findings were suggestive of pyogenic granuloma. Excision of the mass was performed under local anesthesia.

## Discussion

Otorhinolaryngologists, dermatologists, and dental surgeons frequently encounter mass lesions of the oral cavity in their daily clinical practice. While taking the history, it is very important to ask about the duration, onset, progression, and association of these lesions with pain to rule out serious pathology. The etiology may belong to a normal anatomical variant, cyst, inflammatory condition, congenital, or developmental anomaly, and neoplastic or non‐neoplastic conditions. Pyogenic granuloma is one of the causes of soft tissue swelling of the oral cavity that probably results from excessive hyperplasia of the tissue in response to trauma. Low grade trauma such as chronic irritation due to a sharp tooth or a bad technique of tooth brushing may result in excessive tissue repair response, of which the patient may be unaware. This benign vascular neoplasm usually is a few millimeters in size, presenting as a papule or nodule, although cases with sizes up to a few centimeters have also been reported 3. Other synonymously used terms for this condition include lobular capillary hemangioma, telangiectatic granuloma, and “pregnancy tumor” as it has been frequently found in pregnant women.

The exact etiology of pyogenic granuloma is not known. It was thought to be a botryomycotic infection in the past. Currently, the etiopathogenesis of this condition is thought to be related to chronic trauma, which provides a pathway for invasion of the tissue by the nonspecific microorganisms that, in turn, provide stimulus for the excessive proliferation of the vascular type of connective tissue. Granulation tissue thus formed may be covered with fibrin over the surface that mimics pus 4. In our patients, we could not find any specific sources of chronic trauma or irritation. However, both had poor oral hygiene, which might have been the precipitating factor. Pyogenic granuloma most commonly occurs on the gingiva. Extragingival sites include the tongue, hard palate, lip, buccal mucosa, and the floor of the mouth. Among these subsites of the oral cavity, the upper lip is rarely involved. Pyogenic granuloma commonly occurs in the second and fifth decade of life and is more common in females. Both of our patients were females.

The differential diagnosis of oral PGs include hemangioma, lymphangioma, peripheral giant cell granuloma, peripheral ossifying fibroma, conventional granulation tissue, malignancy, Kaposi's sarcoma, angiosarcoma, non‐Hodgkin's lymphoma, syphilis, tubercular ulcer, traumatic ulcer, and cutaneous horn of the lip 5. In our case, the huge PG of the tongue clinically mimicked a “verrucous carcinoma,” posing a diagnostic challenge. Hemangioma and squamous papilloma were also considered as provisional diagnoses. Similarly, the upper lip PG was masquerading as an angiosarcoma. Punch biopsy of the soft tissue lesions for the HPE is useful to confirm the diagnosis or rule out serious pathology that has similar clinical appearance. Our case of PG of the tongue was huge, with a small pedicle, so we performed an excisional biopsy after routine investigation. The presence of capillaries having varying calibers on HPE confirmed the diagnosis of pyogenic granuloma. Lobular capillary hemangioma is a more correct term for this lesion because of these characteristic histopathological features.

Surgical excision is the treatment of choice for PG. Surgical excision of PG of the tongue is usually simple, unlike the PG of the upper lip where the postoperative scar formation can be problematic. It can be prevented by other conservative methods such as cryosurgery and laser excision 6. Simple surgical excision was performed in our case, as we did not have the facility of laser and cryotherapy. While treating such lesions, emphasis on maintenance of oral hygiene should be advised; therefore, dental consultation should be obtained when indicated. This benign vascular neoplasm does not undergo malignant transformation, but it can recur occasionally after surgical excision, commonly in gingival sites. Our patients did not come for follow‐up after surgery as they were from remote places.

## Conclusion

Although oral pyogenic granuloma is not an uncommon cause of soft tissue lesions of the oral cavity, it may have an unusual presentation, posing a diagnostic dilemma to the treating surgeon. It is a benign vascular neoplasm resulting from a hyperactive tissue repair response. Histopathological examination confirms the diagnosis and rules out various soft tissue lesions with similar appearance. Surgical excision is the treatment of choice. Recurrence is rare after surgical excision.

## Conflict of Interest

None declared.

## Authorship

RP: primary author, wrote, structured, formatted case report. SM: critically reviewed the manuscript.
